# Natural Killer Cell Immunotherapy in Solid Tumors: Microenvironmental Obstacles and Translational 3D Models

**DOI:** 10.3390/biology15141167

**Published:** 2026-07-16

**Authors:** Giulia Palazzo, Vincenza Tinnirello, Giulia Bivona, Giulio Ghersi, Simona Campora

**Affiliations:** 1 Department of Biological, Chemical and Pharmaceutical Sciences and Technologies (STEBICEF), University of Palermo, 90128 Palermo, Italy; giuliapalazzo12@gmail.com (G.P.); vincenza.tinnirello@unipa.it (V.T.); giulio.ghersi@unipa.it (G.G.); 2 Department of Biomedicine, Neurosciences and Advanced Diagnostics, University of Palermo, 90127 Palermo, Italy; giulia.bivona@unipa.it; 3 Abiel srl, 90141 Palermo, Italy

**Keywords:** natural killer (NK) cells, solid tumors, tumor microenvironment (TME), extracellular matrix (ECM), immunotherapy, hypoxia and acidosis, spheroids, scaffolds and hydrogels, organ-on-chip

## Abstract

Natural killer (NK) cells represent a promising tool for cancer immunotherapy; however, their efficacy against solid tumors is severely limited by the hostile tumor microenvironment (TME). This review provides a comprehensive overview of the physical, molecular, and metabolic barriers that drive NK cell dysfunction and immune evasion, emphasizing the physical challenge posed by extracellular matrix (ECM) density, which restricts infiltration. Beyond structural barriers, we examine the role of immunosuppressive cytokines (e.g., TGF-β) and immune checkpoint upregulation, both of which directly inhibit NK cell activation. Furthermore, NK cell signaling and cytotoxicity are profoundly affected by metabolic stressors such as hypoxia and acidosis, which act synergistically with the accumulation of immunosuppressive metabolites, including adenosine. These factors impair antitumor activity through multiple mechanisms, particularly the shedding of activating ligands. To investigate these complex interactions, we evaluate the advantages and disadvantages of different three-dimensional (3D) preclinical platforms, including tumor spheroids and Organ-on-Chip technologies, highlighting their distinct characteristics. Rather than advocating for a single technology, we emphasize that each model offers unique advantages for studying specific physical, chemical, and cellular components of the TME. Ultimately, leveraging the capabilities of these advanced 3D platforms is essential for deciphering microenvironmental barriers and unlocking the full therapeutic potential of NK cells against solid tumors.

## Simple Summary

Natural Killer (NK) cells are powerful components of our immune system capable of killing tumor cells. Despite their high potential, their effectiveness in vivo is significantly compromised in solid tumors due to the surrounding tumor microenvironment, which creates physical, molecular, and chemical barriers that block and exhaust NK cells. This review explores these obstacles, highlighting how, unlike two-dimensional in vitro models, advanced three-dimensional models—such as tumor spheroids and Organ-on-Chip—allow for the simulation of these barriers, facilitating the study and promoting the development of more effective immunotherapeutic strategies against solid tumors.

## 1. Introduction: Biology, Regulation, and Cytotoxic Strategies of NK Cells

Natural Killer (NK) cells are effector lymphocytes of the innate immune system that originate from common lymphoid progenitors (CLPs) in the bone marrow. Unlike T and B lymphocytes, which rely on somatic recombination, NK cells use a fixed repertoire of germline-encoded receptors to distinguish between healthy and cancer cells [1]. During maturation, NK cells progressively express receptors such as Ly49, CD11b, CD43, and CD49b, thereby acquiring specialized migratory properties, increased sensitivity to cytokines, and the capacity to produce interferon [2,3]. This maturation process ultimately results in the diversification of NK cells into two main functional subpopulations based on CD56 expression: the immunomodulatory CD56^bright^ population, predominant in lymphoid tissues, characterized by high proliferative capacity and cytokine secretion but comparatively low cytotoxicity; on the other hand, CD56^dim^ population, which comprises the majority of circulating NK cells, distinguished from their counterparts by potent cytotoxic activity mediated by perforin and granzymes and by elevated expression of CD16, which confers high antibody-dependent cellular cytotoxicity (ADCC) [4,5]. Beyond this classic dichotomy, recent single-cell transcriptomic analyses have revealed a complex landscape of tissue-resident NK cells, whose unique receptor profiles and metabolic programs are influenced by specific microenvironmental signals [6,7].

**Figure 1:**
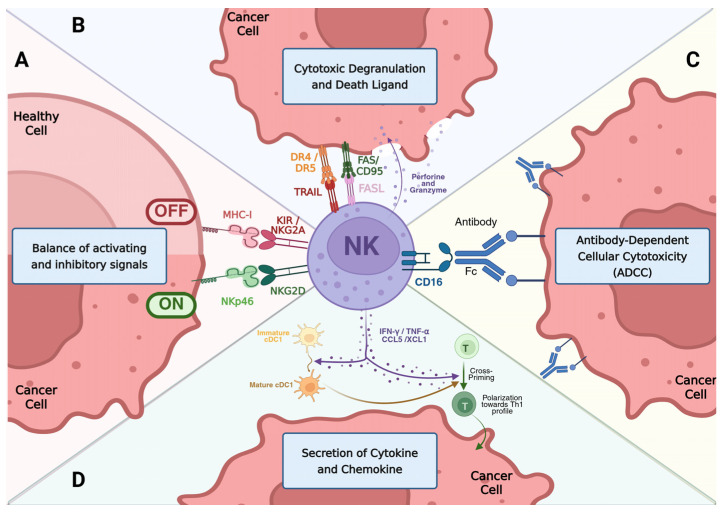
Overview of the mechanisms of recognition, cytotoxicity, and immune crosstalk in natural killer (NK) cells. (**A**) Balance of activating and inhibitory signals: the fate of the NK cell depends on the balance of receptor signals; the binding of activating receptors to ligands on tumor cells triggers the cytotoxic response (“ON” signal), while the interaction between inhibitory receptors and class I MHC molecules on healthy cells preserves self-tolerance (“OFF” signal). (**B**) Cytotoxic Degranulation and Death Ligand: the release of perforins and granzymes induces pore formation in the membrane and the subsequent apoptosis of the tumor cell; in parallel, cell death is mediated by the Fas/FasL axis and TRAIL pathways. (**C**) Antibody-dependent cellular cytotoxicity (ADCC): the CD16 receptor on NK cells recognizes the Fc portion of antibodies bound to tumor antigens, leading to the lysis of the target. (**D**) Secretion of cytokines and chemokines: activated NK cells release soluble mediators that drive the recruitment and maturation of dendritic cells (DCs). DCs, in turn, educate T lymphocytes, promoting, in collaboration with the soluble mediators, their differentiation toward a Th1 phenotype competent against the tumor. Abbreviations: ADCC, antibody-dependent cellular cytotoxicity; DC, dendritic cell; FasL, Fas ligand; NK, natural killer; TRAIL, TNF-related apoptosis-inducing ligand.

The functional fate of an NK cell is governed by a sophisticated balance between activating and inhibitory signals [8]. This balance enables NK cells to rapidly distinguish between healthy and abnormal cells, ensuring an effective response against tumor or infected cells without compromising normal tissues. Inhibitory receptors, such as Killer-cell Immunoglobulin-like Receptors (KIR) and NKG2A/CD94, sustain self-tolerance by recognizing Major Histocompatibility Complex Class I (MHC-I) molecules (HLA-C, HLA-E) on healthy tissues (Figure 1A) [9]. The loss or the reduction in MHC-I expression, a phenomenon frequently observed in cancer cells, removes these inhibitory signals and promotes NK cell activation mediated by activating receptors, such as NKp46 and NKG2D, according to the principle of “missing-self recognition” [10,11].

The cytotoxic activity of NK cells depends on a versatile, finely tuned arsenal that adapts to various pathological conditions through complementary mechanisms: the release of cytotoxic granules, activation of receptor-mediated cell death pathways, and ADCC [12]. The primary and most rapid mechanism is granule exocytosis. After recognizing the target cell, NK cells coordinate the formation of a stable immunological synapse and reorganize their cytoskeleton to direct lytic granules toward the contact site. The exocytosis of perforin forms pores in the target cell’s plasma membrane, allowing granzymes (serine proteases that induce apoptosis via caspase-dependent and -independent pathways) to enter and induce cell death within minutes [13,14].

At the same time, NK cells can activate the extrinsic pathway of apoptosis through the expression of FasL (Fas ligand) and TRAIL (TNF-related apoptosis-inducing ligand). These ligands interact with their respective death receptors (Fas/CD95 and DR4/DR5) on target cells, inducing receptor oligomerization and formation of the DISC (Death-Inducing Signaling Complex), which initiates the apoptotic cascade via caspases 8 and 10 (Figure 1B). The efficacy of this mechanism is finely regulated by the microenvironment and thus by the functional state of the NK cells; soluble factors such as pro-inflammatory cytokines like interleukin-2 (IL-2) and type I interferons such as Interferon-alpha (IFN-α) and Interferon-alpha IFN-β enhance its efficacy, while TRAIL’s ability to act in both membrane-bound and soluble forms ensures considerable flexibility of action, allowing NK cells to induce apoptosis in target cells even in the absence of direct contact [15,16]. Far from being a backup system, the FasL/TRAIL pathway is fully integrated into the cytotoxic strategy and plays a crucial role in serial killing [13,17]. Thanks to efficient synapse recycling and remarkable metabolic flexibility, NK cells can eliminate multiple targets in succession: once their lytic granule reserves are depleted, they continue to exert effector function by using death receptors while the granules are replenished [12]. This property is considered a critical determinant in solid tumors, where high cell density and spatial barriers require prolonged cytotoxic performance. Furthermore, these ligands play a crucial immunoregulatory role in maintaining homeostasis by eliminating hyperactivated immune cells to prevent excessive responses [16]. Finally, a cornerstone of NK cells’ clinical efficacy is ADCC, mediated by the CD16 receptor (FcγRIIIa) (Figure 1C). When IgG antibodies opsonize tumor cells, binding to CD16 triggers massive degranulation and a potent cytotoxic response independent of MHC recognition. This mechanism not only ensures targeted and adaptive elimination but also constitutes the biological foundation for the synergistic use of therapeutic antibodies in oncology [18].

## 2. NK Cells in the Tumor Microenvironment: From Surveillance to Immunomodulation

NK cells represent the immune system’s first line of defense against malignant transformation, as they can recognize and eliminate transformed cells without prior sensitization [19]. However, their role in antitumor immunity is not limited to the simple elimination of transformed cells but extends to a complex surveillance and coordination of the overall immune response. NK cells act as rapid sentinels during the elimination phase of tumor immune editing. Thanks to their ability to recognize the “missing-self” and stress ligands without prior sensitization, they can eradicate unstable cell clones before an immunosuppressive microenvironment is established [12]. A crucial aspect of their role is their ability to selectively target Cancer Stem Cells (CSCs); these populations, which are often resistant to chemotherapy and invisible to T lymphocytes due to low MHC-I expression, exhibit high susceptibility to NK-mediated cytotoxicity, making NK cells essential for preventing tumor recurrence [20,21]. In addition to direct cytotoxicity, NK cells act as “conductors” of the tumor microenvironment. By secreting pro-inflammatory cytokines such as Interferon-gamma (IFN-γ) and TNF-α and chemokines such as CCL5 and XCL1, they promote the recruitment and maturation of conventional type 1 dendritic cells (cDC1): this NK-DC dialogue is essential for the cross-priming of CD8+ T lymphocytes, instructing them to recognize tumor antigens, and for the polarization of the response toward a T-helper 1 (Th1) profile (Figure 1D). This transformation is crucial for converting cold, immunologically silent tumors into hot, immunotherapy-responsive tumors [22]. The protective role of NK cells is particularly evident in preventing metastatic spread; circulating tumor cells (CTCs) are extremely vulnerable to NK cell surveillance in the bloodstream, where the physical barriers of the solid tumor are not yet present. Studies conducted in NK-deficient mouse models (such as the Mcl1^fl/fl Ncr1-Cre model) have confirmed that the absence of these cells leads to a dramatic increase in metastatic colonization, thereby identifying NK cells as the primary guardians against systemic disease progression [23].

Clinical studies have shown that the extent of NK cell infiltration within the tumor mass is positively correlated with a favorable prognosis in numerous solid tumors, including colorectal cancer [24], lung cancer [25], and gastric cancer [26]. This observation has been further corroborated and extended to other tumor types through pan-cancer transcriptomic analyses [27].

Despite the potent antitumor functions described, NK cell activity is often compromised in advanced-stage solid tumors. This transition toward immune failure stems not only from intrinsic defects in NK cells but also from the hostile architecture of the tumor microenvironment, which imposes physical and biochemical barriers often invisible in traditional 2D assays. A comprehensive analysis of these obstacles is therefore essential for understanding the need to employ spatially relevant 3D models that accurately represent the intricate dynamics of NK-tumor interactions.

## 3. The First Obstacle: Accessibility and Penetration in Solid Tumors

The clinical efficacy of NK-cell-based immunotherapies in solid tumors primarily depends on the ability of these effector cells to traverse a physically and chemically hostile environment. In contrast to hematologic malignancies, solid tumors are distinguished by a dense and rigid extracellular matrix (ECM) that acts as a robust physical barrier limiting immune cell infiltration into the tumor mass. Despite their high cytotoxic potential, NK cells often face a logistical challenge in solid tumor environments, notably infiltration difficulties. Clinical and preclinical observations have demonstrated that NK cells remain confined to the peritumoral stroma and are unable to penetrate the tumor mass; this has led to the theorization of an “immune exclusion” hypothesis, which attributes the limited infiltration to the synergistic effects of physical barriers and disrupted chemotactic signals. Consequently, understanding the interplay among ECM architecture, stromal cell activity, and altered chemokine mechanisms that either redirect or retain NK cells at the tumor periphery is crucial for the development of strategies aimed at restoring effective NK cell trafficking and enhancing their antitumor activity.

### 3.1. The Mechanical Barrier: Matrix Stiffness

The extracellular matrix of solid tumors undergoes profound remodeling driven by cancer-associated fibroblasts (CAFs), which secrete large amounts of type I and IV collagen. This process is mediated by enzymes such as lysyl hydroxylase 2 (LH2) and lysyl oxidase (LOX), which cause hyper-cross-linking of fibers [28], leading to a drastic increase in tissue stiffness, from values below 1000 Pa (<1 kPa) in healthy tissues to 4–10 kPa in tumors [29,30], and decreased matrix porosity. This remodeled ECM not only impairs the diffusion of nutrients and therapeutic agents but also forms a physical barrier that substantially restricts immune cell infiltration [31].

**Figure 2:**
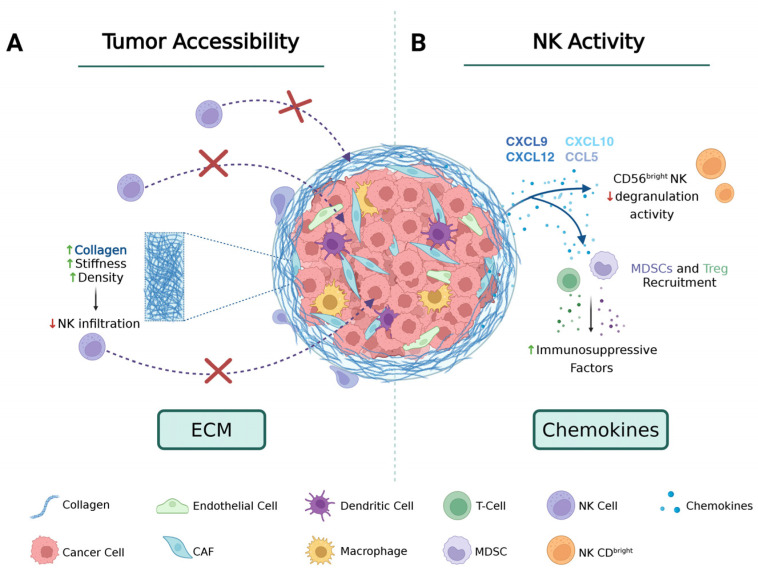
Physical and chemokinetic barriers of the tumor microenvironment (TME) that regulate the infiltration and activity of natural killer (NK) cells. The figure illustrates the cellular heterogeneity of the TME—composed of tumor cells, endothelial cells, cancer-associated fibroblasts (CAFs), dendritic cells, and macrophages—and its impact on NK cell migration. (**A**) Tumor accessibility: collagen accumulation and remodeling increase the density and stiffness of the extracellular matrix (ECM); this structural barrier physically blocks NK cell passage, preventing their penetration and infiltration into the tumor core. (**B**) NK activity: the chemokine gradient in the TME (CXCL9, CXCL10, CXCL12, and CCL5) alters immune recruitment. It promotes the selective attraction of the CD56^bright^ NK cell subset, characterized by reduced degranulation and cytotoxic capacity; on the other hand, it drives the recruitment of myeloid-derived suppressor cells (MDSCs) and regulatory T cells (Treg), which help maintain a local immunosuppressive niche by releasing inhibitory factors. Abbreviations: CAFs, cancer-associated fibroblasts; ECM, extracellular matrix; MDSCs, myeloid-derived suppressor cells; NK, natural killer; Treg, regulatory T cells.

The physiological collagen network, which is porous and randomly oriented, transforms into a dense, linearized architecture, demonstrating a significant reduction in interstitial pore size from 10–50 μm in normal tissues to 1–10 μm in tumors [32]. Since NK cells rely on amoeboid migration, which requires pores of adequate size and low mechanical resistance, they exhibit particular sensitivity to these biophysical constraints among all cytotoxic lymphocytes. This suggests that the spatial configuration of the extracellular matrix is a critical factor in immune exclusion. Experimental evidence indicates that NK cells tend to accumulate preferentially in stromal regions rich in fibroblasts and with high collagen concentrations, due to physical barriers that block their infiltration into the tumor mass, thereby creating an obstacle that NK cells are unable to overcome (Figure 2A) [33]. Furthermore, the stiffness of the matrix and the aligned architecture of the collagen fibers do not merely constitute a physical barrier but also induce true phenotypic remodeling of NK cells. This effect is mediated by the activation of surface integrins that are sensitive to mechanical stress [30,34]. Their signaling triggers the selective downregulation of primary activator receptors such as NKG2D, DNAM-1, and CD16 [35], leading to decreased cytotoxic activity and causing NK cells that manage to infiltrate by overcoming the ECM barrier to exhibit a poorly cytotoxic phenotype [36]. These findings are further supported by computational modeling studies, which provide mechanistic insights and demonstrate that highly aligned collagen fibers, typical of advanced-stage tumors, limit immune cell access and produce models of immune exclusion that are consistent with clinical observations [37].

Taken together, these findings suggest that collagen deposition, cross-linking, and fiber alignment constitute significant obstacles to NK cell infiltration in solid tumors, underscoring the need for ECM-targeted strategies to improve the effectiveness of NK cell-based immunotherapies.

### 3.2. Chemokine Diversion: How Tumors Redirect NK Cell Traffic

In addition to the extracellular matrix, which acts as a physical barrier to NK cell infiltration, the tumor microenvironment contains soluble components, such as cytokines and chemokines, that interfere with the directional signals required for proper NK cell function. Instead of guiding the most cytotoxic NK subpopulations towards malignant tissue, tumors manipulate chemokine expression to attract less effective populations and to repel those with the highest antitumor potential [38].

CD56^dim^ NK cells, recognized as the primary mediators of tumor lysis, are characteristically enriched for receptors responding to inflammatory chemokines such as CX3CR1, CXCR1 and CXCR2. These receptors mediate responses to ligands such as CX3CL1, CXCL1 and CXCL8 (IL-8), making the cells particularly responsive to concentration gradients typical of inflamed tissues [39,40]. However, in solid tumors, the recruitment of this cytotoxic fraction is hindered in two ways: on the one hand, the local production of these inflammatory chemokines is frequently reduced or halted; on the other hand, immunosuppressive factors such as TGF-β reduce CX3CR1 expression on CD56^dim^ cells, impairing their extravasation and reducing recruitment [41]. At the same time, the tumor microenvironment frequently exhibits elevated levels of chemokines associated with IFN-induced or homeostatic responses, including CXCL9 and CXCL10 (via the CXCR3 axis), as well as CXCL12 (via the CXCR4 axis), and chemokines such as CCL5 [42] (Figure 2B). Because CD56^bright^ NK cells constitutively express high levels of CXCR3 and CXCR4 [43], this chemokine profile selectively recruits this less cytotoxic subset into the tumor microenvironment. Concurrently, these signals attract immunosuppressive populations, such as macrophages, myeloid-derived suppressor cells (MDSCs), regulatory T cells (Treg), and other cell types that secrete immunosuppressive factors and modulate the extracellular matrix. This process amplifies the diversion of immune traffics, subsequently impeding the infiltration and functional activity of cytotoxic NK cells [44].

In addition to conventional circulating NK cells (cNK), tissue-resident NK cells (trNK) are a crucial and highly dynamic component of the tumor microenvironment. TrNKs constitute a distinct lineage that develops and matures directly in peripheral tissues, acquiring surface retention markers such as CD69, CD49a, CD103, and CXCR6, along with transcriptional programs and functions shaped by the local microenvironment [45,46,47]. Unlike circulating cNKs, most physiological trNKs exhibit reduced basal cytotoxicity, with lower expression of perforin and granzymes, a higher activation threshold, and a predominantly immunomodulatory profile oriented toward tissue homeostasis [48,49].

In the context of cancer, the tumor microenvironment exploits the marked plasticity of trNKs, conditioning their function through persistent exposure to local immunosuppressive factors [45,48]. This conditioning drives trNKs toward a hypofunctional or dysfunctional “resident-like” phenotype, characterized by expression of inhibitory receptors such as TIGIT, TIM-3, NKG2A, and PD-1 [45,49], and by marked suppression of direct degranulation and IFN-γ secretion, thereby promoting an environment that facilitates tumor progression [46,49].

However, trNKs play a markedly ambivalent role in the antitumor response. Although their direct lytic capacity is attenuated, recent studies show that intratumoral trNKs can act as key organizers of adaptive immunity: by secreting chemokines such as XCL1, XCL2, and CCL5, trNKs selectively recruit conventional type 1 dendritic cells (cDC1) within the TME [6,46]. This cDC1–trNK axis promotes antigen presentation and facilitates the infiltration and activation of CD8+ T lymphocytes, converting immunologically “cold” microenvironments into reactive ones [6,48]. Consistent with this positive immunomodulatory role, the presence of a transcriptional signature associated with trNKs correlates with improved overall survival in multiple solid tumors, including pancreatic ductal adenocarcinoma (PDAC) [6,48] and lung cancer [49]. Nevertheless, under the continuous pressure of tumor cytokines, the drive toward dysfunction often prevails.

Consequently, even when NK cells infiltrate the tumor, the composition of the infiltrate, skewed toward CD56^bright^ cells and functionally reprogrammed trNK cells, tends to be less effective at tumor control because the cells present are not the most suitable for direct lysis [41]. This chemokine-mediated reconfiguration is multifactorial: it results from tumor- and stroma-derived ligand production, proteolytic activity that alters ligand availability, and cytokine modulation that changes receptor expression on NK cells; plasticity induced by interleukins IL-2 and IL-15, as well as transforming growth factor-beta (TGF-β), can lead to increases or decreases in specific receptors. Experimental studies also demonstrate that local restoration of favorable chemokines (for example, CCL5) or engineering NK cells to express corresponding receptors enhances infiltration and antitumor efficacy in preclinical models [50].

## 4. The Second Barrier: Molecular and Metabolic Inactivation in the Tumor Microenvironment

Even if the NK cells recognize the tumor site, they encounter an additional challenge: a highly hostile microenvironment that induces their functional inactivation. Upon infiltrating the tumor, NK cells undergo a progressive transition towards exhaustion, driven by exposure to inhibitory signals and metabolic stress [51]. Their cytotoxic activity is compromised by stringent molecular regulation exerted by suppressive cytokines, such as TGF-β [52], and by the expression of immune checkpoints [53]; concurrently, NK cells undergo a process of metabolic paralysis due to critical conditions, including hypoxia, acidosis, and the accumulation of immunosuppressive metabolites [54]. Understanding how the tumor orchestrates this inactivation of NK cells is essential for the development of multimodal strategies aimed at restoring NK cell effector functions and reprogramming the immune response within solid tumors.

### 4.1. Molecular Brakes: TGF-β Signaling and Immune Checkpoints

Once NK cells infiltrate the tumor, they are subjected to a sophisticated network of molecular inhibitory mechanisms that diminish cytotoxic functionalities and induce a state analogous to exhaustion. Among these, transforming growth factor beta (TGF-β) plays a significant role: it has been extensively studied over the years for its multifaceted functions in various physiological processes and its involvement in multiple diseases such as chronic inflammation, fibrosis, and cancer [55].

**Figure 3:**
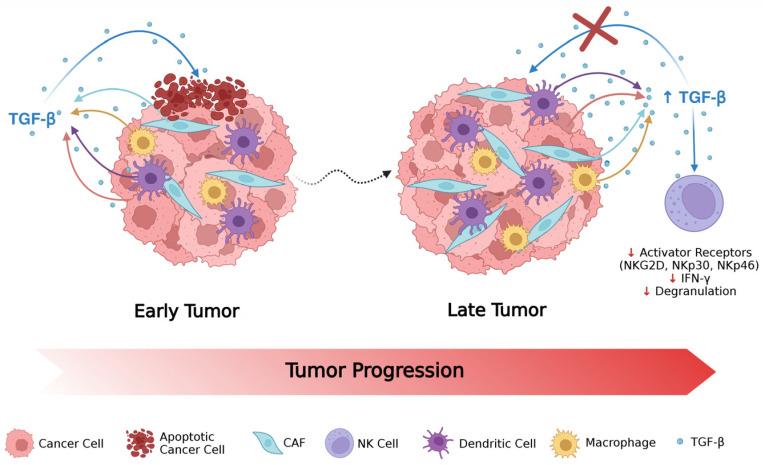
The dual role of TGF-β during tumor progression and its impact on the plasticity and evasion of NK cells. The timeline illustrates the functional switch of TGF-β secreted by components of the tumor microenvironment (tumor cells, CAFs, dendritic cells, and macrophages) as the tumor progresses from an early-stage lesion to an advanced stage. Early tumor: TGF-β secreted into the microenvironment primarily exerts an oncosuppressive effect, inducing cell cycle arrest and apoptosis (represented by apoptotic cells/cell death) in tumor cells. Late tumor: as the disease progresses, tumor cells become resistant to the pro-apoptotic signals of TGF-β. Consequently, the overproduction and massive accumulation of this cytokine mediate profound immunosuppression that specifically targets infiltrating NK cells. At the molecular level, chronic exposure to TGF-β in advanced tumors leads to downregulation of key activation receptors (NKG2D, NKp30, NKp46), a drastic reduction in interferon-gamma (IFN-γ) production, and blockade of cytotoxic degranulation mechanisms. Abbreviations: CAF, tumor-associated fibroblasts; IFN-γ, interferon-gamma; NK, natural killer; TME, tumor microenvironment.

In oncological contexts, TGF-β is distinguished by its pleiotropic role, modulated by its dual functionality and complex cellular origin (Figure 3). In the initial phases of tumorigenesis, TGF-β exerts a potent tumor-suppressive action by promoting cell cycle arrest and apoptosis [56,57]. An example of this protective role has been observed in breast cancer, where BMP7, a member of the TGF-β family, inhibits human telomerase (hTERT), leading to telomere shortening and subsequent cell death [58].

However, the boundary between the suppressive and pro-tumor functions of TGF-β is marked by the progressive accumulation of genetic and epigenetic aberrations in advanced tumor cells. Mutations or deletions in SMAD4, the primary receptors TGFBR1 and TGFBR2, and downregulation of the type III co-receptor (TGF-βIII), together with the epigenetic silencing of key genes such as SMAD2 or the overexpression of endogenous repressors of the pathway, such as Smad7, Ski, and SnoN, disrupt the canonical signaling pathway [59]. This block prevents formation of the functional Smad2/3–SMAD4 complex, thereby inhibiting the subsequent transcription of antiproliferative genes, such as p15 and p21, and the repression of proliferative genes, such as c-Myc.

In addition to classical transcriptional regulation, recent evidence shows that SMAD4 plays a key tumor-suppressive role by modulating tumor metabolism. For example, in ovarian cancer, SMAD4 regulates the transcription of ARHGAP10, thereby suppressing glycolysis induced by the PI3K/AKT/HK2 pathway [60]. Loss of SMAD4 or its receptors disrupts these metabolic and transcriptional checkpoints, triggering an oncogenic “switch” in which the tumor acquires resistance to the suppressive action of TGF-β. In this context, integrating SMAD4 loss with hyperactivation of non-SMAD pathways, such as MAPK/ERK, JNK, p38, and PI3K/AKT, radically reprograms the biological output of TGF-β [61] transforming it into a powerful driver of epithelial–mesenchymal transition (EMT), angiogenesis, and metastatic aggressiveness, as evidenced by the association between elevated levels of TGF-β1 and clinical progression in breast and prostate cancers [56,57,58]. This temporal transition radically redefines the microenvironment: while TGF-β inhibits tumor growth in the early stages, in the advanced stages the resistance acquired by neoplastic cells transforms the cytokine into a purely immunosuppressive factor. This functional paradox is supported by a broad secretory network that does not involve tumor cells alone. In fact, TGF-β is also actively produced by cancer-associated fibroblasts (CAFs), myeloid-derived suppressor cells (MDSCs), tumor-associated macrophages (TAMs), and dendritic cell subpopulations. This heterogeneous pool of sources ensures a high local concentration of TGF-β, which, by creating an immunosuppressive gradient, actively inhibits the metabolic fitness, degranulation, and cytotoxic activity of NK lymphocytes, significantly compromising the host’s immune surveillance [59].

TGF-β-mediated attenuation of NK cell activity occurs through a synergistic, multilayered inhibitory mechanism. At the metabolic and transcriptional levels, TGF-β antagonizes IL-15-induced mTOR kinase activation—a central hub for metabolic reprogramming and glycolysis in NK cells—by suppressing expression of the transcription factors T-bet and Eomes [62]. This leads to the downregulation of primary activating receptors such as NKG2D, NKp30, and NKp46, and a concurrent reduction in effector molecules such as perforin, granzyme B, and IFN-γ [63]. This transcriptional and metabolic block reprograms cellular networks toward tissue-resident cell signatures or undifferentiated ILC1-like and decidual-like phenotypes, marked by a drastic loss of antitumor function and, at times, the acquisition of pro-angiogenic properties in the neoplastic microenvironment [62,63].

In addition to direct suppression, TGF-β acts synergistically with the immune checkpoint network: exposure to TGF-β induces increased expression of inhibitory receptors such as NKG2A [64], TIGIT [65], TIM-3 [66], and, in certain contexts, Programmed Death-1 (PD-1) [67]; concurrently, tumors upregulate the corresponding ligands (HLA-E, CD155, PD-L1), thereby forming inhibitory synapses that diminish degranulation and cytokine secretion. This molecular coordination creates an inhibitory synergy: TGF-β deprives NK cells of activation signals and hypersensitizes them to immunological brakes mediated by inhibitory synapses, accelerating the transition toward a phenotype of functional exhaustion. Preclinical and translational research indicates that blockade of specific checkpoints (e.g., anti-NKG2A or anti-TIGIT) can partially restore NK effector functions, thereby supporting the application of checkpoint inhibition as part of NK-targeted therapeutic strategies [68,69].

Finally, recent evidence shows that prolonged exposure to TGF-β imprints this dysfunctional profile on NK cells in a stable manner through persistent epigenetic remodeling. The loss of chromatin accessibility at IRF, T-bet, and Eomes, along with a reduction in the active histone modification H3K4me3 at the promoters of effector genes, durably suppresses cytotoxicity even after the signal is removed, as also observed in clinical contexts such as hepatocellular carcinoma [70].

Canonical TGF-β signaling proceeds via the TGFβR—SMAD2/3 pathway with the formation of nuclear complexes with the SMAD4, which modulates transcriptional programs in NK cells and in tumor/stromal compartments. Translational studies provide evidence that disrupting the SMAD axis can release NK cells from TGF-β-mediated suppression: CRISPR-mediated deletion of SMAD4 in human NK cells preserves cytotoxicity, cytokine secretion, and tumor infiltration across various NK cell platforms, suggesting an engineering strategy to generate TGF-β-resistant NK cells for adoptive transfer [71]. At the same time, murine genetic studies show that SMAD4 plays both TGF-β-dependent and independent roles in NK maturation and homeostasis, implying potential trade-offs between achieving resistance to suppression and maintaining normal developmental programs, which must be evaluated when designing SMAD4-targeting approaches [72].

To neutralize this complex inhibitory network, several targeted therapeutic strategies have been developed that combine molecular interception of TGF-β with cell engineering and checkpoint blockade.

At the systemic pharmacological level, TGF-β neutralizing monoclonal antibodies (such as Fresolimumab) [73] or TGF-βR1 receptor kinase inhibitors (such as Galunisertib) [74] have been shown to attenuate desmoplasia and restore NK cell function. Furthermore, recent bifunctional fusion proteins (TGF-β Traps), including Bintrafusp alfa (M7824)—composed of the ectodomain of the TGF-βRII receptor fused to an anti-PD-L1 antibody—sequester TGF-β directly within the tumor microenvironment while simultaneously blocking the PD-1/PD-L1 axis, thereby specifically overcoming synergistic inhibition [75,76].

In the field of adoptive cell therapy, genetic engineering offers advanced solutions to mitigate the risks of toxicity associated with systemic TGF- blockade. In addition to SMAD4 editing [71], the expression of a dominant-negative TGF-β receptor (dnTGF-β RII)—lacking the intracellular kinase domain—allows CAR-NK cells to act as molecular “decoys,” making them inherently resistant to immunosuppressive signals from the TME [77,78].

Tumor epigenetic programs may also enhance the molecular brake circuit. The inhibition or loss of the histone methyltransferase EHMT2 in tumor cells reduces TGF-β1 production, increases expression of NKG2D ligands and chemokines that promote NK cell recruitment, and restores NK-dependent tumor control in preclinical models, thus establishing a connection between tumor epigenetics and NK evasion while identifying an upstream node that is amenable to pharmacological targeting [79].

In summary, to overcome the marked inhibitory synergy observed, clinical efficacy will require a combined multimodal therapeutic approach. This strategy must be structured simultaneously around the protection or genetic modification of NK cells to make them resistant to intrinsic TGF-β signaling—either through SMAD4 editing or through the expression of dnTGF-βRII receptors—the use of neutralizing antibodies or molecular traps (TGF-β Traps) to reduce the availability of the soluble cytokine in the TME, and finally on the combined application of antibodies targeting induced immune checkpoints, such as anti-NKG2A and anti-TIGIT, to dismantle active inhibitory synapses.

Preclinical evidence supports each of these approaches; however, translational development must carefully consider biological context (for instance, the roles of SMAD4 in maturation), potential compensatory pathways, and the risks linked to systemic blockade of TGF-β [71,72,79].

### 4.2. Metabolic Paralysis: Hypoxia and Acidosis

The infiltration of NK cells into the tumor mass exposes them to additional metabolic stress, which rapidly compromises their cytotoxic efficacy. Within the tumor microenvironment, a dense extracellular matrix and abnormal vascularization decrease oxygen supply, creating a hypoxic environment that activates hypoxia-inducible factors, particularly Hypoxia-Inducible Factor 1-alpha (HIF-1α), which, upon stabilization, dimerizes with HIF-1β to initiate transcriptional programs aimed at tumor survival [80,81]. This process upregulates the expression of critical glycolytic genes (e.g., GLUT1, LDHA), pro-angiogenic factors (e.g., vascular endothelial growth factor, VEGF), and genes involved in survival pathways and cellular plasticity, thereby contributing to an aggressive phenotype and promoting the formation of aberrant vascularization that sustains the hypoxic state [82,83,84].

**Figure 4:**
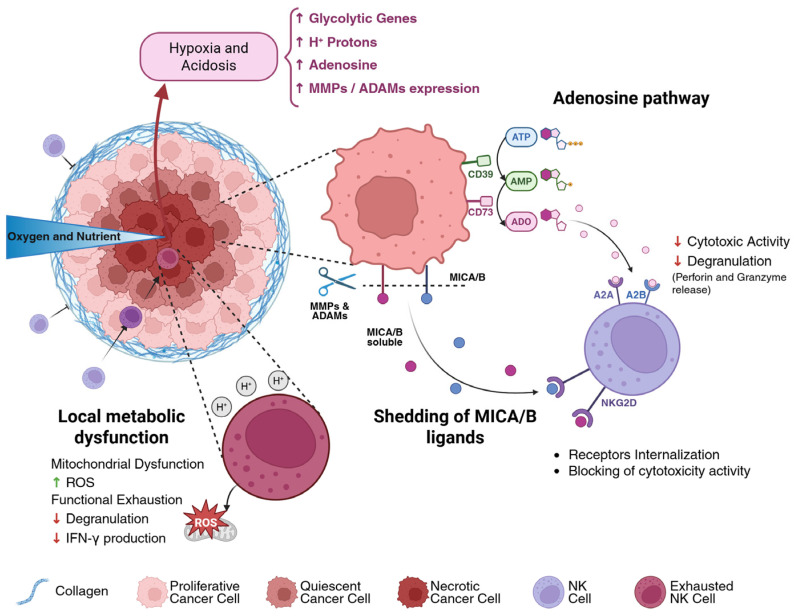
Systemic effects of hypoxia and acidosis on TME metabolism, mitochondrial dysfunction in NK cells, and evasion mechanisms mediated by adenosine (ADO) and soluble MICA/B. Hypoxia and acidosis induce upregulation of glycolytic genes, proton (H+) extrusion, adenosine accumulation, and overproduction of proteases (matrix metalloproteinases, MMPs and A Disintegrin and Metalloproteinase, ADAMs). The tumor mass exhibits a chromatic gradient toward the interior, reflecting decreasing gradient of oxygen and nutrients that culminate in a hypoxic and acidic core. The dense surrounding collagen matrix impedes access by most NK cells. Local metabolic dysfunction: the few NK cells capable of infiltrating the mass are affected by H+ accumulation; local acidosis induces mitochondrial dysfunction and a massive increase in reactive oxygen species (ROS), depleting degranulation capacity and IFN-γ production. Adenosine pathway: The enlarged tumor cell illustrates the activity of the ectoenzymes CD39 and CD73, which sequentially convert extracellular ATP into AMP and, ultimately, into adenosine (ADO). The binding of ADO to the A2A and A2B purinergic receptors expressed on NK cells suppresses their cytotoxic and degranulatory activity. Shedding of MICA/B ligands: MMP and ADAM proteases cleave MICA/B ligands from the tumor surface, converting them into a soluble form. Soluble MICA/B binds to the NKG2D activator receptor on NK cells, inducing its internalization and consequent blockage of cytotoxic capacity. Abbreviations: ADAM, a disintegrin and metalloproteinase; AMP/ATP, adenosine monophosphate/triphosphate; ADO, adenosine, IFN-γ, interferon-gamma; MMP, matrix metalloproteinase; NK, natural killer; ROS, reactive oxygen species.

From an immunological perspective, hypoxia and HIF-1α signaling promote the metabolic reprogramming of NK cells, favoring a shift towards glycolysis at the expense of oxidative phosphorylation, which results in mitochondrial dysfunction, characterized by increased levels of reactive oxygen species (ROS) and a loss of effector functions, such as degranulation and IFN-γ production (Figure 4) [85,86]. Concurrently, HIF-1α enhances immunosuppression by elevating the expression of matrix metalloproteinases (MMPs) and other factors that facilitate the shedding of activating ligands, thereby stimulating pathways that recruit suppressive myeloid cells, and promoting the accumulation of metabolites (lactate, adenosine) that further impair NK cell functionality [87].

Closely associated with hypoxia is the development of an acidic environment (pH ≤ ~6.5) resulting from excessive lactate production and proton accumulation characteristic of the Warburg effect [88,89]. This state of acidosis impairs NK cell motility, thereby restricting their infiltration [90,91]; it hinders the formation of cytotoxic synapses and reduces the sensitivity of activating receptors, it leads to the accumulation of immunosuppressive metabolites such as adenosine and lactate, which suppress activating signals via metabolic receptors (A2A/A2B) and promote the induction of metabolic checkpoints [92].

The mechanisms described are interconnected: hypoxia enhances tumor glycolysis and lactate production, thereby decreasing pH and intensifying mitochondrial damage in NK cells, establishing a vicious cycle that perpetuates the metabolic paralysis. From a translational perspective, these findings support adopting multimodal approaches to restore NK cell function: vascular normalization and improved oxygenation to optimize therapeutic perfusion windows; metabolic preconditioning or NK cell engineering aimed at augmenting mitochondrial biogenesis and oxidative capacity (e.g., modulation of Peroxisome proliferator-activated receptor gamma coactivator 1-alpha, PGC-1α or selection of stress-resistant subpopulations); and targeted approaches against immunosuppressive metabolites through A2A receptor antagonists, lactate dehydrogenase (LDH) inhibitors, or CD39/CD73 inhibitors, supplemented by local pH buffering techniques [82,92].

Experimentally, three-dimensional and microfluidic models that recreate O_2_ and pH gradients are essential for evaluating NK cell penetration and function, while functional endpoints such as CD107a, real-time cytotoxicity, IFN-γ, and metabolic assessments assist in correlating phenotype with metabolic activity [93].

### 4.3. Evasion and Mimicry Strategies: The Role of Adenosine and Ligand Shedding

Hypoxia-induced metabolic remodeling not only compromises NK-cell metabolism directly but also establishes the biochemical conditions that sustain additional immune escape mechanisms. Among these, extracellular adenosine accumulation and protease-mediated shedding of activating ligands represent two complementary strategies by which tumors convert metabolic stress into impaired immune surveillance.

In the tumor microenvironment, elevated adenosine levels are associated with the enzymatic activities of CD39 and CD73, which facilitate tumor progression and the formation of metastases via the PI3K/AKT signaling pathway [94,95,96] and contribute to establishing an immunosuppressive environment [97]. Adenosine generated by the AMP hydrolyzing enzymes CD39 and CD73 interacts with A2A and A2B receptors on NK cells, impairing their cytotoxic function, reducing their capacity to release perforin and granzymes, and hindering intercellular interactions (Figure 4) [98,99,100].

Simultaneously, a complementary strategy adopted by tumors involves the proteolytic shedding of activating ligands (MICA/MICB) facilitated by proteases from the A Disintegrin and Metalloproteinase (ADAM) and MMP families. The soluble ligands accumulate within the microenvironment and circulation, saturating activating receptors, particularly NKG2D, and thereby promoting internalization or functional desensitization of NK cells. This process impairs direct recognition and destabilizes the cytotoxic synapse, ultimately contributing to poorer prognoses across various malignancies [101,102]. As demonstrated in the studies conducted by Ferrari De Andrade et al., preclinical inhibition of shedding, achieved through the application of antibodies specific to the α3 domain of MICA and MICB, leads to a significant increase in the density of stimulatory ligands on the surface of tumor cells, resulting in the restoration of the activating pathway facilitates the elimination of tumor cells by NK cells [103]. Similar results were reported by the research team led by Courau et al., involving heterotopic spheroids, where the administration of antibodies directed against MICA/MICB enhanced the immune-mediated destruction of tumor spheroids, inducing an increase in NK cell infiltration and activation [104]. These mechanisms do not operate independently but are interconnected and mutually reinforcing: adenosine signaling promotes an immunosuppressive stroma that elevates proteolytic activity and ligand shedding, while the reduction in membrane ligand expression increases NK cell reliance on chemokine-guided trafficking, a process often subverted by tumors to exclude cytotoxic NK subpopulations [47].

The most promising therapeutic strategies are both combinatorial and targeted. These approaches include A2A and A2B receptor antagonists or CD39 and CD73 inhibitors to block adenosine signaling; ADAM and MMP inhibitors or ligand-stabilizing antibodies to prevent shedding and maintain NKG2D engagement; and chemokine-based interventions, such as ex vivo engineering of chemokine receptors or localized chemokine administration, to enhance NK cell homing and retention within the tumor microenvironment. Three-dimensional preclinical models that preserve soluble factor gradients and ligand release are particularly useful for evaluating these therapeutic combinations [93,105].

## 5. Three-Dimensional Models for the Study of NK Cell Immunotherapy

Due to the critical role of the tumor microenvironment in regulating NK cell anticancer activity, in vitro immune studies require highly complex systems such as three-dimensional (3D) models. The use of 3D platforms to study NK cells—whose cytotoxic activity and infiltrative capacity are intricately influenced by the complex architecture of the tumor microenvironment—is no longer merely a methodological choice, but an essential requirement to ensure the translational relevance of the obtained data [106].

The implementation of 3D culture systems stems from the necessity to bridge the disparity between two-dimensional (2D) in vitro assays and in vivo animal models. For decades, 2D cell culture on rigid plastic substrates has served as the primary model in preclinical research due to its simplicity, reproducibility, and cost-effectiveness. However, this model creates a highly artificial environment for the cells, compelling them to adhere to a flat surface, which alters their morphology, polarity, and, consequently, the organization of their cytoskeleton. The absence of a third dimension eliminates the physicochemical gradients of oxygen, nutrients, and metabolites characteristic of the tumor microenvironment in vivo, thereby significantly diminishing its complexity [107]. This discrepancy has historically led to an overestimation of the efficacy of drugs and immunotherapies [108,109]. Therefore, the transition to 3D systems constitutes a crucial evolution, as it facilitates cell–cell and cell-ECM interactions, enhances extracellular vesicle (EV) secretion and promotes the production of EV cargo that more closely resembles that of in vivo systems under physiological conditions, thereby incorporating features that are absent in 2D cultures [110]. In the specific case of NK cells, in addition to reproducing the structural complexity of solid tumors, 3D models enable the quantitative assessment of immune-specific functional readouts that are difficult to capture in conventional 2D cultures.

Crucially, these platforms enable researchers to actively engineer and reproduce the distinct pathophysiological features of the tumor mass, such as extracellular matrix density, chemokine gradients, immunosuppressive factor accumulation, hypoxia, and localized acidosis. By mimicking these in vivo barriers in a highly controlled manner, 3D systems provide a realistic testing ground to evaluate how the physical, molecular, and metabolic target structures discussed in Section 3 and Section 4 dynamically suppress NK cell infiltration and cytotoxicity.

Furthermore, 3D models permit to investigate the NK cell infiltration depth, migration dynamics, immune synapse formation, cytotoxic activity, target-cell killing kinetics, and the acquisition of exhaustion-associated phenotypes through functional and molecular analyses. These immune-specific functional readouts, together with the corresponding quantification strategies, are discussed in detail in the following subsections dedicated to each 3D platform.

The main features of the different 3D models are summarized in Table 1.

### 5.1. Scaffold-Free Tumor Spheroids and Organoids

Among three-dimensional models, spheroids, derived from single-cell suspensions, are the most widely used system owing to their ease of generation and reproducibility. Comprising dense cell aggregates capable of growing in suspension, these models emulate the oxygen and nutrient gradients characteristic of solid tumors in vivo, facilitating the development of a central hypoxic and necrotic core [133]. The application of spheroids in the investigation of NK cells holds particular significance, as it enables the assessment of cytotoxic efficacy not merely as an immediate event, as observed in 2D cultures, but as a function of penetration depth [134,135]. Consequently, NK cells encounter a critical temporal challenge: they must infiltrate the tumor mass and perform their function before microenvironmental conditions adversely affect their phenotype, thereby diminishing their cytotoxic capabilities. Real-time imaging of NK cell cytotoxicity within these systems unveils dynamic patterns of cellular killing, dose-dependent responses, and sensitivities specific to tumor lineage, thereby enabling quantification of infiltration, immune synapse formation, and the spatial distribution of cell death [136,137].

However, because of their homotypic nature, numerous spheroids do not facilitate a comprehensive investigation of stromal interactions. To address these limitations, more sophisticated models, specifically Multicellular Tumor Spheroids (MTSs) or Assembloids, have been developed. These are aggregates composed of varied cell lines, typically tumor cells, endothelial cells and fibroblasts, combined in fixed, consistent proportions, thereby enhancing the model’s diversity and more accurately replicating the complexity of actual tumor masses. Notably, the incorporation of fibroblasts not only promotes the synthesis of a natural collagen-rich ECM, rendering assembloids ideal tools for investigating how stromal density affects NK cell infiltration, but also plays a pivotal role in specific pathways associated with the inhibition of NK cytotoxic activity [138,139], such as the increased production of TGF-β [140]. Experiments conducted by Leontova et al. on heterotopic spheroids demonstrated that the extracellular matrix encasing the tumor mass functions as a substantial barrier to NK cell infiltration; these cells tend to become confined within fibroblast-rich stromal regions, thereby failing to reach their natural target of the tumor cells [33]. In perfect concordance with these findings, recent research conducted by Lo Cicero et al. confirmed the restrictive role of the ECM in the penetration of chemotherapeutic agents [141]. In their study, the use of targeted enzymes—such as recombinant collagenases of class I and class II—to degrade the collagen component of the matrix drastically reduced the structural rigidity of the ECM, promoting the entry and diffusion of therapeutic molecules like doxorubicin into the treated spheroids compared with the control [141,142].

Furthermore, these 3D models offer the possibility to simultaneously analyze the migration, invasion, and subsequent antitumor activity of immune cells as suggested by Sherman et al. using transwell systems [143].

On the other hand, organoids offer an additional level of biological fidelity. Unlike assembloids, organoids are typically derived directly from patient biopsies and cultured in such a way that the various cellular components organize themselves spontaneously, without adhering to rigid proportions imposed by the researcher [144]. This self-organization process produces highly variable models that substantially diminish the model’s reproducibility, while preserving the genetic and phenotypic characteristics of the original tumor. Analyses conducted on long-term organoid cultures suggest that the characteristics of the original tumor sample, such as phenotype, genetic diversity, and mutational signatures, are faithfully maintained in the organoids [145,146].

Studies conducted on organoids have been utilized to assess the specificity and cytotoxicity of engineered NK cells (chimeric antigen receptor (CAR)-engineered NK, CAR-NK) in vitro. Furthermore, by employing live confocal imaging techniques, it has been feasible to dynamically observe recruitment, migration, infiltration, local density, and killing kinetics at the single-organoid level, thereby providing valuable data to enhance current understanding and optimize immunotherapeutic strategies [147].

However, traditional tumor organoid models focus primarily on the neoplastic and stromal components, largely neglecting the intricate complexity of the immune microenvironment. A major breakthrough is represented by patient-derived immunocompetent tumor organoids (PDTOs), which actively integrate cellular immune components. Advanced platforms utilizing submerged cultures in Matrigel and air-liquid interface (ALI) methods—based on the pioneering protocols of Neal and Kuo [148]—3D microfluidic systems and 3D bioprinting enable the structural preservation of endogenous tumor-infiltrating immune populations or the precise incorporation of autologous immune cells, vascularization, and CAFs, successfully recreating a dynamic tumor ecosystem in vitro [149,150]. In particular, these immunocompetent systems have recently been used to link phenotype to function through ex vivo drug screening, directly predicting treatment sensitivity and personalized immune response, as successfully demonstrated in bladder cancer models [151].

The translational and predictive value of PDTOs is currently being optimized through the integration of cutting-edge technologies in the field of spatial omics, a methodological branch that has undergone a profound evolution since the first spatial transcriptomics techniques described by Ståhl [152] and subsequent refinements in high-resolution analysis of the tumor microenvironment, led by Longo [153]. The use of these advanced spatial approaches, which currently include spatial transcriptomics, proteomics, and metabolomics, is essential for accurately assessing how faithfully these 3D architectures replicate the molecular organization and cellular arrangement in vivo [154,155].

While real-time imaging physically tracks NK cell migration, spatial omics map the entire molecular profile while preserving the organoid’s 3D spatial coordinates. This regional approach allows researchers to decode the spatial interactome and cell–cell communication patterns at single-cell resolution, identifying localized cellular niches and distinguishing the different transcriptomic and metabolic signatures that emerge between the periphery and the hypoxic core of the organoid [154,155]. Consequently, this approach reveals the precise signals of juxtacrine and paracrine inhibition that are activated at the exact moment an infiltrating NK cell encounters immunosuppressive niches populated by CAFs or TAMs.

Despite this considerable potential, the application of spatial omics to 3D models still faces numerous methodological challenges, including transcript diffusion, non-uniform cell adhesion, and persistent technical difficulties in consistently achieving single-cell resolution [155]. For these reasons, the field of patient-derived organoids is undergoing a transition toward translational applications. Analysis of registered clinical trials shows that, while standard PDOs are already being successfully used in prospective oncology studies to guide therapeutic decisions, immunologically complex platforms combined with spatial multi-omic profiles remain primarily limited to the preclinical phase, due to technical, logistical, and ethical barriers that hinder their large-scale clinical adoption [151].

Although they are more complex to manage, organoids represent the most advanced platform for personalized medicine, enabling a more accurate assessment of NK cell infiltration and cytotoxic activity within a structure that faithfully reflects each patient’s clinical profile.

### 5.2. Scaffolds and Hydrogels: Mimicking the ECM

The tumor microenvironment plays a central role in interactions between immune cells and the tumor. Acquiring models that facilitate a more comprehensive investigation of this component is, therefore, crucial. In this context, a notable advancement in 3D models involves the incorporation of spheroids or organoids within hydrogel matrices of various types and compositions to vary porosity and fiber density, aims to replicate the tumor stroma more accurately and support targeted studies [156].

The selection of matrix is critical in determining the biomechanical characteristics of the model. The materials employed are chiefly categorized into natural matrices, such as Matrigel [157], collagen [158], fibrin, and alginate [159,160], which offer a bioactive but less precisely controlled environment; and synthetic matrices, such as Polyethylene glycol (PEG), Polylactic acid (PLA), Poly lactide-co-glycolide (PLG), and poly(lactic-co-glycolic acid) (PLGA) [161], which permit more accurate modulation of stiffness and porosity [159,162]. Therefore, the porosity of the hydrogel affects the mobility and migration velocity of NK cells [163]. The study conducted by Temples et al. not only demonstrates how hydrogel integration provides optimal models for studying the physical barriers encountered by NK cells on their way to the tumor but also stabilizes chemokine and cytokine gradients produced by the spheroid, resulting in a model capable of replicating the chemotactic processes observed in vivo [122]. Consequently, these “hybrid” systems serve as an essential intermediary between the spheroids and the complexity of in vivo systems, providing an optimal platform for evaluating strategies aimed at enhancing lymphocyte recruitment, such as the application of matrix-degrading enzymes or the genetic modification of NK cells with chimeric receptors (CAR-NK) optimized for tissue motility.

Although these models can reconstruct specific 3D structures and partial organ functions in vitro, they still do not entirely replicate the intricate in vivo environment. The lack of vascular systems, neural networks, and immune systems in organoids restricts their utility in studies involving complex physiological processes and multi-tissue interactions.

### 5.3. Organ-on-Chip Systems

Organ-on-chip (OoC) platforms overcome the limitations of static models by simulating perfused, spatially organized tumor microenvironments through the integration of endothelial channels, 3D matrices, and controlled flow. Compared to individual spheroids or organoids, these systems provide a more comprehensive platform that is better suited for investigating intricate dynamic processes such as immune cell migration and extravasation [164,165]. Due to the inclusion of perfusable vascularization and the establishment of stable chemotactic gradients, these platforms enable the simulation of immune cell recruitment from circulation and facilitate the quantification of dynamic parameters such as migration velocity, the duration of effector cell-target cell interactions, and tissue penetration depth through live-imaging microscopy techniques [166].

The integration of OoC systems with organoids allows for the preservation of a patient’s tumor heterogeneity while concurrently incorporating vascular and stromal components; moreover, these elements can be bio-printed within the chip to attain highly specific localization. This methodology enables assessment of how ECM variables, flow dynamics, and stromal cells synergistically modulate NK cell migration and cytotoxic efficacy [164,165].

By enabling physical separation between the “circulating” and “tissue” compartments and the application of physiological shear stress, these platforms provide a fluid-dynamic context for testing cellular and immunomodulatory therapies [36,167]. From a functional perspective, the use of tumor-on-chip technologies has enabled studies of how the tumor’s immunosuppressive microenvironment affects NK cell activity, including NK cell exhaustion. It has been observed that this impairment of cytotoxic capacity persists for an extended period even after the removal of NK cells from the microfluidic platform, suggesting a lasting phenotypic reprogramming of these immune cells, mediated by the tumor microenvironment [168]. A significant example of these applications is the study by Marzagalli et al., who used a microfluidic device to characterize counterflow NK cell extravasation, demonstrating preferential recruitment of low-cytotoxic subpopulations toward neuroblastoma spheroids [36].

These platforms, integrated with adjustable matrices and quantitative pipelines, bridge the gap between static 3D models and in vivo studies, enhancing preclinical predictability for cell therapies and immunotherapies while requiring attention to standardization and experimental scalability [167].

## 6. Conclusions

NK cells are a key component of the immune surveillance system against tumors, thanks to their innate ability to recognize and eliminate cancer cells without prior sensitization. However, despite promising in vitro results, their therapeutic efficacy in vivo against solid tumors remains severely limited. The primary cause is the complex network of immune evasion strategies orchestrated by the tumor microenvironment (TME), which impedes NK cell activity on multiple levels, starting with biochemical and physical accessibility.

The first obstacle is an ECM characterized by high density and rigidity due to massive collagen deposition; this dense structural barrier physically blocks NK cell entry, trapping them in the peripheral stromal region and preventing them from penetrating the tumor core. This barrier not only limits cell traffic but also compromises the diffusion and accessibility of essential activating cytokines (such as IL-2 and IL-15), depriving NK cells of the signals needed for survival and proliferation. In addition to the physical barrier, the TME profoundly alters cell recruitment through abnormal chemokine gradients; elevated expression of CXCL9, CXCL10, CXCL12, and CCL5 favors the preferential recruitment of CD56^bright^ NK cell subpopulations—characterized by high cytokine production but low cytotoxic capacity—to the detriment of CD56^dim^ NK cells, which possess the true therapeutic and killing potential. At the same time, the secretion of soluble immunosuppressive factors such as TGF-β acts directly on infiltrating NK cells, downregulating the expression of their key activating receptors and severely compromising degranulation and the release of perforin and granzymes. Finally, from a metabolic standpoint, NK cells undergo a functional shutdown: the tumor’s dysfunctional vasculature creates steep gradients of oxygen and nutrients, resulting in a hypoxic tumor core with a high rate of metabolic acidity. This hostile environment induces a state of metabolic exhaustion that permanently shuts down the antitumor activity of NK cells.

In this context, the transition from 2D to 3D culture systems represents a crucial advance, providing models that faithfully replicate this architectural complexity. 3D models, such as homotypic and heterotypic spheroids, are essential platforms for observing how the tumor alters the phenotype of NK cells, inducing a state of exhaustion that remains invisible in less complex systems. Spheroids have shown that NK cell efficacy depends heavily on their energy state in hostile environments, where uneven distributions of oxygen and nutrients promote immune suppression. The integration of patient-derived organoids is integral to personalized medicine, as it preserves tumor heterogeneity and each patient’s unique genetic profile, facilitating the development and testing of individualized therapies. Furthermore, integrating these models into hydrogel scaffolds or matrices enables in-depth study of NK cell infiltration within the tumor mass, supporting targeted efforts to overcome physical barriers. A further advance is achieved by integrating 3D models into organ-on-chip platforms, enabling precise simulation of interactions among tumors, stroma, and immune cells and dynamic investigations of NK cell migration and extravasation within tumor models. However, significant translational challenges still hinder the translation of these systems and therapies into clinical and industrial applications. While engineered counterparts such as CAR-NK cells still face challenges related to limited in vivo persistence, insufficient expansion, and functional exhaustion within suppressive tumor niches, the platforms used to model them face major technical bottlenecks, such as the difficulty in standardizing patient-derived organoids due to inherent biological variability, the difficulty of reproducing the full complexity of immune and stromal components, and the high fabrication and operational costs of organ-on-chip technologies, which limit their scalability.

Looking ahead, overcoming the physical and immunological barriers imposed by the tumor microenvironment (TME) requires therapeutic strategies that go beyond simply blocking soluble checkpoints, focusing instead on the intrinsic reprogramming of the effector cell. In this context, a major clinical frontier is represented by the use of cytokine-induced memory-like NK cells (CIML). Ex vivo pre-activation of NK cells with a specific cytokine cocktail, typically consisting of IL-12, IL-15, and IL-18, [169] induces stable memory-like responses, characterized by marked persistence in vivo, high metabolic fitness, and massive IFN-γ production following subsequent tumor stimulation [170,171]. This functional robustness is closely linked to the emerging concept of trained immunity in NK cells [172]. Through stable epigenetic modifications, such as chromatin remodeling and histone acetylation, and metabolic reprogramming oriented toward glycolysis and oxidative phosphorylation, these cells maintain a long-term primed state [173]. The adoption of memory-like NK cells and the design of protocols aimed at inducing trained immunity are emerging as crucial tools for generating resilient cell populations capable of resisting functional exhaustion and effectively penetrating the immunosuppressive microenvironment of solid tumors, paving the way for next-generation, highly personalized cellular immunotherapies [174].

In conclusion, although each 3D system has its own inherent advantages and limitations, their integration provides a more comprehensive understanding of the role of the tumor microenvironment. This represents a pioneering area of research with significant implications for the success of immunotherapies, effectively bridging the gap between in vitro systems and clinical reality. Future efforts should focus on frontier strategies, including metabolic reprogramming approaches aimed at enhancing NK cell fitness and persistence in hostile tumor niches, as well as the strategic co-delivery of biomaterials and hydrogel-based systems capable of physically protecting and biochemically supporting immune cells at the tumor site. Only through the synergistic integration of bioengineering, computational analysis, and molecular immunology will it be possible to decipher the mechanisms underlying tumor resistance and optimize NK cell-based immunotherapies.

## Figures and Tables

**Table 1:** Comparison of different 3D in vitro models for studying NK cell.

3D Model	Key Components	Benefits for NK Cell Research	Limits	References
**Spheroids** 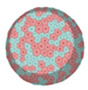	Cell aggregates (omo/heterotypic)	Cytotoxicity analysis in gradients of O_2_, pH and metabolites	Overly simplified stromal architecture; failure to replicate the high density of the real tumor matrix	[111,112,113,114,115]
**Patients derived organoids (PDO)** 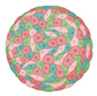	Patient-derived cells (preservation of heterogeneity)	High biological fidelity; study of immune checkpoints and immune evasion mechanisms	High inter-sample variability; lack of protocol standardization and strict ethical/logistical constraints	[116,117,118,119,120,121]
**Scaffold and hydrogel** 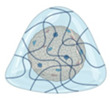	Natural or synthetic matrices	Analysis of the impact of ECM stiffness and density on migration	Static model; inability to replicate dynamic recruitment from the circulation	[122,123,124,125,126]
**Organ-on-Chip** 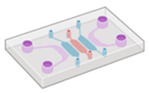	Microfluidics; pseudo-vascular channels; controlled flow	Real-time dynamic study of extravasation, shear stress, and recruitment	High engineering complexity and costs; limited scalability for high-throughput drug screening	[127,128,129,130,131,132]

## Data Availability

No new data were created or analyzed in this study. Data sharing is not applicable to this article.

## Abbreviations

The following abbreviations are used in this manuscript:

NK	Natural killer
TME	Tumor Microenvironment
ECM	Extracellular matrix
3D	Three-dimensional
CLPs	Common lymphoid progenitors
ADCC	Antibody-dependent cellular cytotoxicity
KIR	Killer-cell Immunoglobulin-like Receptors
MHC-I	Major Histocompatibility Complex Class
FasL	Fas ligand
TNF	Tumor necrosis factor
TRAIL	TNF-related apoptosis-inducing ligand
DISC	Death-Inducing Signaling Complex
IL-2	Interleukin 2
IFN-α	Interferon-alpha
DCs	Dendritic cells
CSCs	Cancer Stem Cells
IFN-γ	Interferon-gamma
cDC1	Conventional type 1 dendritic cells
Th1	T-helper 1
CTCs	Circulating tumor cells
CAFs	Cancer-associated fibroblasts
MDSCs	Myeloid-derived suppressor cells
Treg	Regulatory T cells
cNK	Circulating NK cells
TGF-β	Transforming growth factor-beta
hTERT	Human telomerase
trNK	Tissue-resident NK cells
EMT	Epithelial–mesenchymal transition
TAMs	Tumor-associated macrophages
PD-1	Programmed Death-1
HIF-1α	Hypoxia-Inducible Factor 1-alpha
VEGF	Vascular endothelial growth factor
ROS	Reactive oxygen species
MMPs	Matrix metalloproteinases
PGC-1α	Peroxisome proliferator-activated receptor gamma coactivator 1-alpha
LDH	Lactate dehydrogenase
ADAMs	A Disintegrin and Metalloproteinase
ADO	Adenosine
EV	Extracellular vesicle
2D	Two-dimensional
PDO	Patients derived organoids
MTSs	Multicellular Tumor Spheroids
CAR	Chimeric antigen receptor
PEG	Polyethylene glycol
PLA	Polylactic acid
PLG	Poly lactide-co-glycolide
PLGA	Poly(lactic-co-glycolic acid)
OoC	Organ-on-chip
PDTOs	Patient-derived immunocompetent tumor organoids
ALI	Air-liquid interface
CIML	Cytokine-induced memory-like NK cells
